# Favourably regulating two-phase flow regime of flow boiling HFE-7100 in microchannels using silicon nanowires

**DOI:** 10.1038/s41598-021-89466-z

**Published:** 2021-05-27

**Authors:** Tamanna Alam, Wenming Li, Wei Chang, Fanghao Yang, Jamil Khan, Chen Li

**Affiliations:** 1 Department of Mechanical Engineering, University of South Carolina, Columbia, SC 29210 USA; 2 Princeton Plasma Physics Laboratory, Princeton, NJ 08540 USA

**Keywords:** Mechanical engineering, Characterization and analytical techniques

## Abstract

High performance miniaturized electronic devices require enhanced, compact and reliable thermal management system. As an efficient compact space cooling technique, flow boiling in microchannels has recently gained wide acceptance. However, weak buoyancy effects and microgravity in avionics and numerous space systems operations hinder the performance of flow boiling microchannel thermal management system due to poor bubble departure capacity and unfavorable development of flow regimes. Here we report the flow boiling silicon nanowires (SiNWs) microchannels which can favorably regulate two-phase flow regimes by enhancing explosive boiling, minimizing bubble departure diameter, and smoothing flow regime transition. Extensive experimental investigations along with high speed visualizations are performed. The experiments are performed with the dielectric fluid HFE-7100 in a forced convection loop for wide range of heat and mass fluxes. High speed flow visualizations have been employed at up to 70 k frames per second (fps) to understand the boiling mechanism in terms of bubble dynamics, flow patterns, and flow regime developments for SiNWs microchannels. These studies show that SiNWs reduce intermittent flow regimes (slug/churn), improve rewetting and maintain thin liquid film at wall. Therefore, flow boiling in SiNW microchannels is promising to thermal management owing to its high heat transfer rate with low pressure drop and negligible microgravity sensitivity.

## Introduction

Boiling fluid can dissipate significantly higher heat flux utilizing the latent heat of fluid with a smaller flow rate compare to single-phase counterpart. Hence, flow boiling in microchannels has emerged as a promising and effective method to control and minimize high heat fluxes in compact spaces^1, 2^ and it has received increased attention by the researchers in recent years. It has been considered as the most effective heat dissipation method for various high heat density applications. These applications include next generation electronics (3D ICs)^3, 4^, battery packs and inverters/power electronics of electric vehicles/hybrid electric vehicles (EV/HEV)^5–7^, avionics operations and numerous space systems (e.g. power systems, thermal control systems, and life support systems)^8, 9^. However, flow boiling in microchannels encounters challenges due to weak or reduced buoyancy effects in space applications and terrestrial systems like military/avionics operations. In addition, microgravity is not favorable to enhance two-phase flow boiling due to the difficulty in detaching bubbles from heated surface and the disappearance of natural convection.

Several researchers have investigated the effects of gravity in two-phase flow boiling microchannels and reported poor gravitational independency. Simoneau and Simon^10, 11^ studied the forced convection boiling of nitrogen in vertical channel and observed lower critical heat flux (CHF) for down-flow as compared to up-flow test at the same low liquid velocity. At higher liquid velocity, the difference in CHFs for the up-flow and down-flow were decreased due to the diminishing effect of buoyancy. Similar studies have been performed by Mishima and Nishihara^12^ in a rectangular channel (2.4 mm × 40.0 mm) for up-flow and down-flow at low to high velocity ranges. CHF was triggered very quickly at low flow rates for both the up-flow and down-flow cases due to flooding. At high flow rates, CHF was enhanced for up-flow cases due to the establishment of annular flow regime, whereas, CHF was even lower than the low flow rate experiments for down-flow case due to the stagnation of bubble (force balance between drag and buoyancy). Gersey and Mudawar^13, 14^ reported the effect of orientation on CHF during flow boiling experiments. They showed the lowest CHF values for downward facing chips (180°, − 135°, and − 90°) for liquid velocities below 200 cm/s resulting from vapor stagnation followed by dryout. Zhang et al.^15^ theoretically and experimentally studied the effects of orientation on flow boiling CHF. They used high-speed video and micro-photographic techniques to reveal a fairly continuous wavy vapor layer which travel along the heated wall and permit liquid contact only in wetting fronts, located in the troughs of the interfacial waves. CHF initiated when wetting fronts near the outlet were lifted off the wall. Kandlikar and Balasubramanian^16^ investigated an effect of gravitational orientations on flow boiling characteristics of water in a set of six parallel minichannels. The individual channel dimension is 1054 μm wide, 197 μm deep, and 63.5 mm long with a hydraulic diameter of 333 μm. Three orientation namely, horizontal, vertical down-flow, and vertical up-flow were investigated under identical operating conditions of heat and mass fluxes. Their experiments with high-speed flow visualization studies indicated that the flow became less chaotic for the vertical up-flow case compared to the horizontal case, while the reversed flow became more pronounced in the vertical down-flow case. Therefore, a gravity insensitive flow boiling system is urgently needed to enhance nucleate boiling and evaporation as well as to passively introduce advection by inducing high frequency liquid renewal on walls.

Recently, nanowires (NWs)^17, 18^ and carbon nanotubes (CNTs)^19–21^ were used in different studies to enhance the nucleate pool boiling and convective boiling in microchannels^19, 22–26^. An enhanced heat transfer coefficient and higher critical heat flux were reported due to the higher nucleation site density and the enhanced wettability respectively. A novel boiling surface with engineered submicron pores (formed by NW bundles) which are surrounded by nanoscale pores (created by individual NWs) were developed earlier by our team^27^. The aim of this work was to address NASA’s needs that is especially favorable for applications in microgravity by creating a new, unified and ultra-efficient flow boiling pattern in two-phase technologies. This SiNW microchannel has the ability to minimize the transitional flow boiling regimes, i.e., slug/churn/wavy patterns to a single annular flow starting from onset of nucleate boiling (ONB) to CHF condition by guiding the flow structure in two aspects: reducing bubble size and changing the direction of the surface tension force from the cross-sectional plane to the inner-wall plane^29, 30^. The effects of gravity in flow boiling SiNW microchannels system using deionized water have been studied and no/little influences in SiNW microchannels have been observed as reported in our earlier studies^28, 29^. In addition, an enhanced heat transfer and CHF limit with reduced pressure drop and flow boiling instabilities were observed from these studies using deionized water as working fluid^27, 30, 31^.

**Figure 1:**
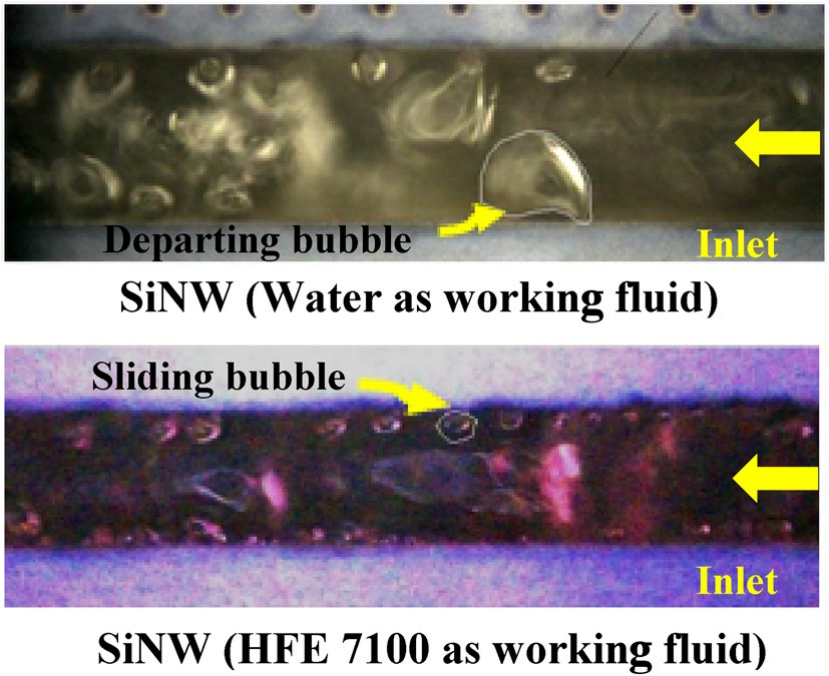
Bubble growth mechanism in flow boiling SiNW microchannels using water and HFE-7100 as working fluids.

It is well accepted that deionized (DI) water is the best among all the two-phase coolants, especially for its high latent heat of vaporization (2260 kJ/kg). Significant improvement of heat transfer performances along with gravity insensitivity have been observed in flow boiling SiNW microchannels using deionized water compare to plainwall microchannels^27–31^. However, due to its relatively high saturation temperature of 100 °C at 1 atm., high corrosive properties and polarization capacity, its suitability for application as coolant for electronic chips is very much debated. Using dielectric fluids with lower saturation temperature is a viable alternative. Major thermo-physical properties of HFE-7100 (1 atm. and 25 °C) are given in Table 1. The latent heat of evaporation for dielectric fluids is generally almost an order of magnitude lower than that for water and literatures report poor heat transfer performances. Additionally, unique thermo-physical properties of HFE-7100, such as low surface tension and high vapor density compared to water, could significantly alter the transport phenomena. Fluid with low surface tension reduces the capillary pressure, and tends to blow away from the heating walls by vapor flow and fails to form favourable thin liquid film on the walls for desirable heat transfer performance. Moreover, high vapor density could impact on bubble dynamics and may show higher gravity sensitivity characteristics. A significant difference in bubble growth and departure mechanism between water and HFE-7100 as working fluid can be seen from Fig. 1 from our recent ongoing studies. Hence, further studies are needed to investigate the flow boiling characteristics in SiNW microchannels using dielectric fluids.

**Table 1:** Major thermos-physical properties of HFE-7100 (1 atm., 25 °C).

Properties	Values
Latent heat of vaporization (kJ/kg)	112
Specific heat (kJ/kg K)	1.18
Boiling point (°C)	61
Liquid density (kg/m^3^)	1510
Kinematic viscosity (cSt)	0.38
Surface tension (mN/m)	13.6
Vapor pressure (kPa)	26.8

In this study, we have performed experimental investigation with dielectric fluids, HFE 7100, for a wide range of heat and mass flux conditions to evaluate the performance of flow boiling SiNW microchannels and access its applicability for practical applications. In addition, high speed flow visualization up to 70,000 fps has been performed to explore the flow boiling mechanism, flow regime development and bubble dynamics. Furthermore, SiNW experimental data have been plotted in different established flow regime map (Taitel-Dukler map^32^, Ullmann–Brauner^33^, Harirchian and Garimella^34, 35^) to better understand the observed flow patterns during visualization studies. Finally, the results are compared with plainwall microchannels.

## Methods

### Experimental setup

**Figure 2:**
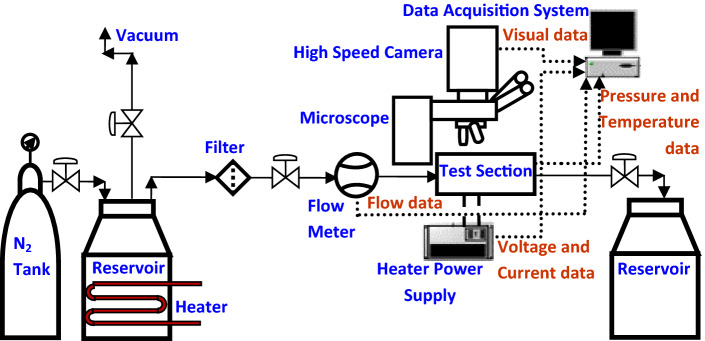
Schematic diagram of flow loop with high speed camera and data acquisition system^40^.

An experimental flow loop, an optical high-speed imaging system and a data acquisition unit are the three major components of the experimental setup as shown in Fig. 2. In the experimental flow loop, working fluid (pumped by compressed nitrogen, N_2_) from a pressurized working fluid reservoir passed through an inline filter, an Omega flow meter (0.03 kg/m^2^s resolution), and microchannel test section and finally returned to an outlet reservoir. A heater-vacuum system was used to degas the working fluid prior to the experiments. Electrical power was supplied by a high precision digital programmable power supply and micro-heater voltage was measured by an Agilent digital multimeter. The inlet and outlet fluid temperature were measured using two Omega K type thermocouples and the inlet and outlet pressure of the test section were measured using a pressure transmitter. Two high speed flow visualization cameras namely, Vision Research Phantom v7.3 and Vision Research Phantom v711 were linked to an optical microscope (Olympus microscope, BX-51 with 400 × amplifications) to capture images for the flow boiling in SiNW microchannels at frame rate ranges 4000–70,000 fps. Flow rate, inlet and outlet pressure, inlet and outlet temperature, and voltage and current data were recorded by a customized data acquisition system developed from National Instrument (NI) LabVIEW. The schematic diagram of flow loop, high speed imaging system and data acquisition system are shown in Fig. 2. More details of the experimental setup are available in our previous study^27, 40^.

### Experimental test section

**Figure 3:**
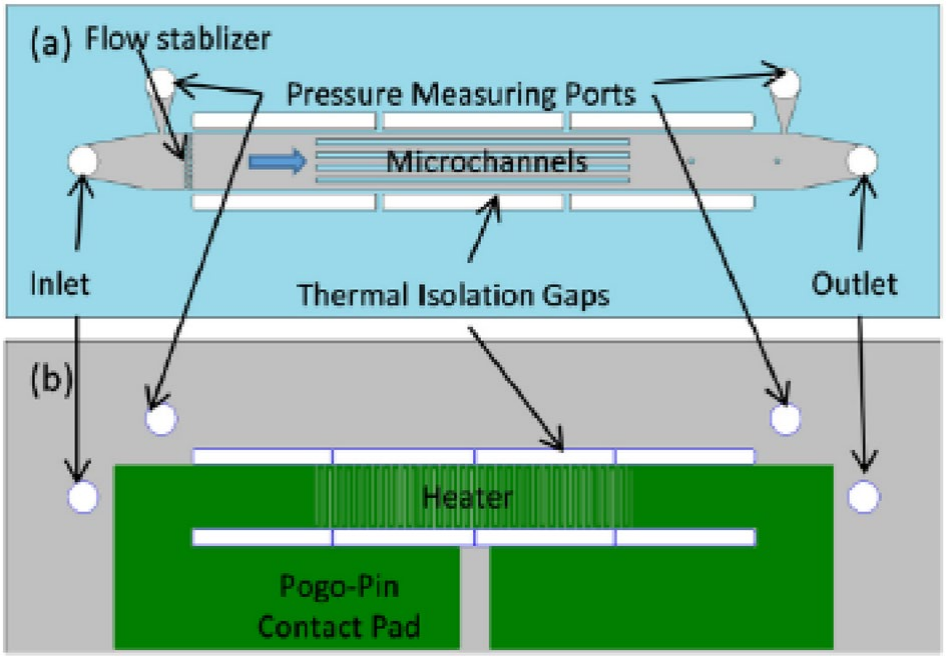
Micro devices configurations (**a**) front-side and (**b**) back-side of the microchannel device^27^.

Microchannel test section assembly contains a copper housing with inlet and outlet plenum, test chip and pressure transducers and thermocouples at inlet and outlet of the test chip. Microchannel test chip consists of five parallel straight microchannels and each channel dimension is width, *w*: 220 μm × height, H: 250 μm × length, L: 10 mm. The microchannel test chip is made from a silicon wafer bonded to a Pyrex wafer by a standard microfabrication process. Micro devices configurations are shown in Fig. 3. The design and fabrication of the microchannel devices were described in our previous study^27^.

**Figure 4:**
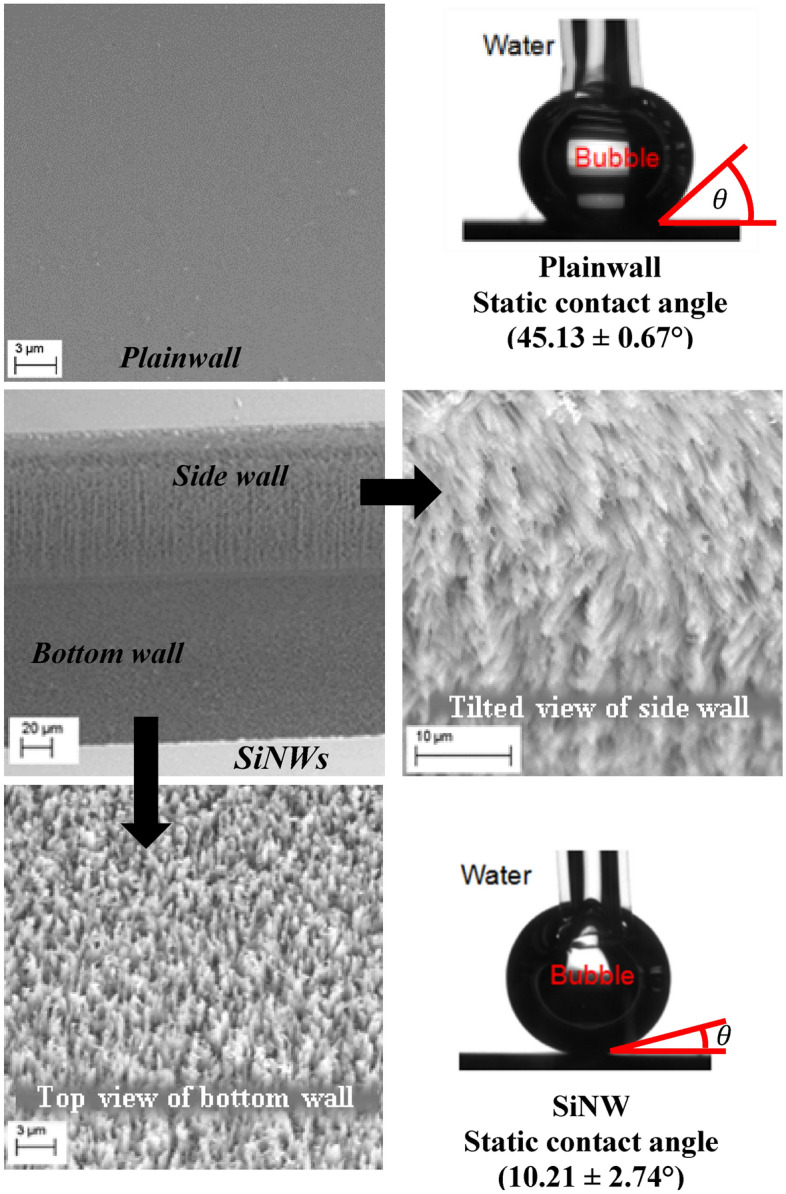
Scanning electron microscope (SEM) images of Plainwall and SiNW surfaces with measured static contact angles.

A resistor, which serves as both a micro heater to generate heat flux and a thermistor to measure the wall temperature, is deposited onto the back side of the silicon chip. The heating area (length, L: 10 mm × width, W: 2 mm) is identical to the total base area of microchannel arrays. The silicon nanowire surface was produced in smooth silicon surface by electroless electrochemical etching technique with silver nanoparticles (AgNPs) catalyst. These SiNWs were then naturally oxidized to make super hydrophilic surface (approximate contact angle 0º) using the Wenzel effect^36, 37^. Representative scanning electron microscope (SEM) images of Plainwall and SiNW surfaces with measured static contact angles are shown in Fig. 4. SiNWs are approximately 20–100 nm in diameter and 5 µm in length with nearly uniform coating over the boiling surfaces including bottom and side walls as can be seen in the figure. SiNWs form nanoscale to submicron scale pores between nanowire bundles and these pores work as nucleation cavity to enhance nucleation site density. Details of the fabrication process of micro-device were reported in our earlier studies and can be found in our previous published work^27^.

### Calibration

The thermistor was calibrated in an isothermal oven and a relationship (confidence of the correlation coefficient ≈ 0.9999) between micro-heater temperature and it’s electric resistance was generated using a linear curve fitting prior to the experiments. The heat loss from the microchannel test section was evaluated experimentally and expressed as a function of temperature difference between micro device and ambient temperature. The curve obtained by linear fitting was used to estimate heat loss with a high accuracy^38^.

### Test procedure

In these flow boiling studies, experimental investigation in SiNW microchannels with dielectric fluids, HFE 7100 have been performed for a wide range of mass fluxes, from 400 to 1600 kg/m^2^s. During the experiments, for a fixed flow rate condition, a uniform heat flux was provided to the chip in steps by a digital power supply through the micro-heater until approaching CHF conditions. For each data point, the data acquisition system recorded 120 sets of steady state experimental data including voltages, currents, local pressures and temperatures at inlet and outlet at 4 min intervals. Additionally, high speed flow visualization was performed from the top of the microchannel test section for each condition. More descriptions of the flow loop, test device and experimental details can be found in our previous studies^38, 39^. Repeatability of the experimental data has been carried out for different mass fluxes, heat fluxes, orientations and test samples extensively for our current test setup. The measurement accuracies and experimental uncertainties associated with sensors and parameters can be found in the supplementary file.

## Results

### Differences in flow pattern transition in Plainwall and SiNW microchannels

**Figure 5:**
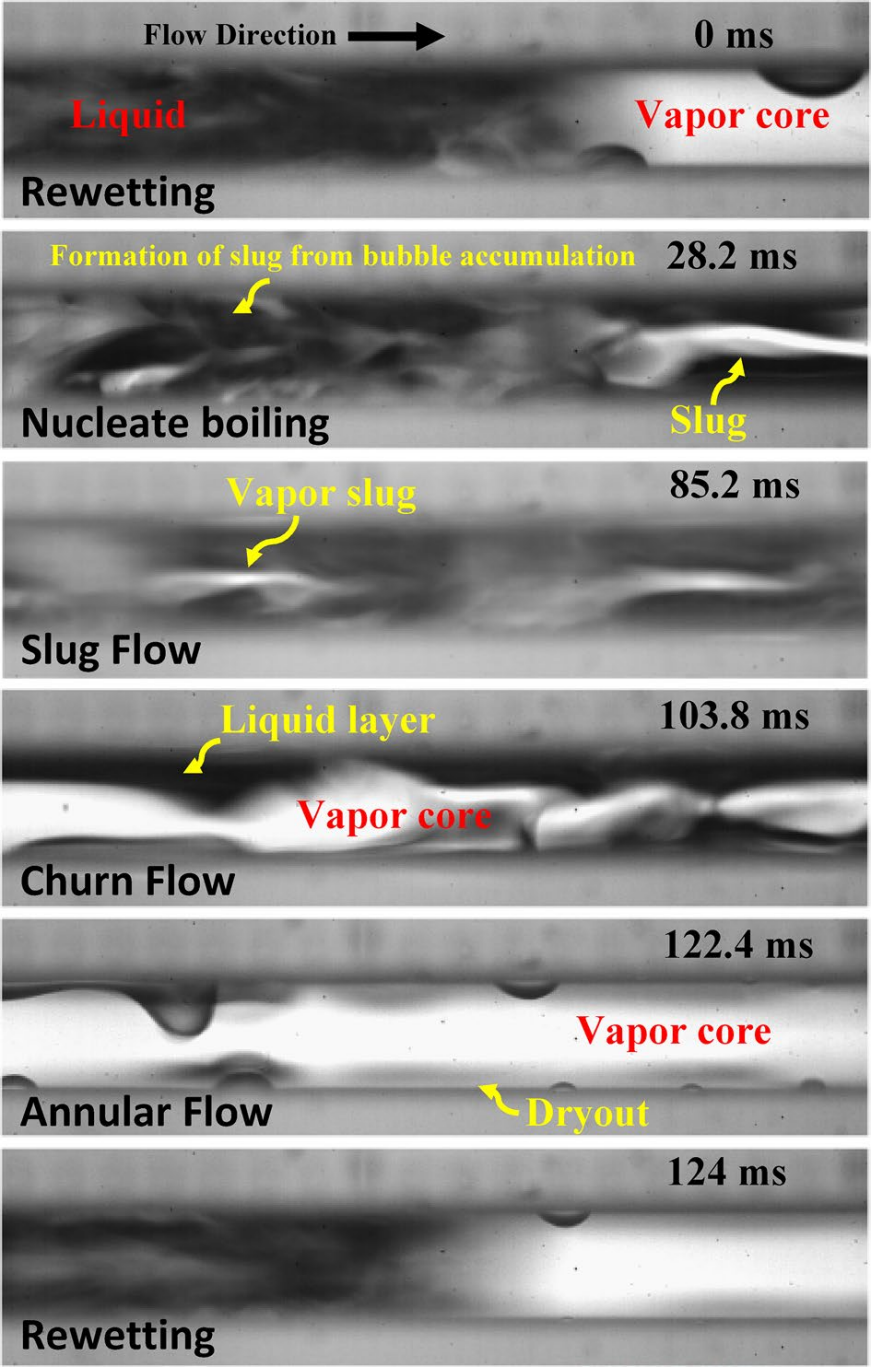
Flow pattern transitions in flow boiling plainwall microchannels at mass flux, 400 kg/m^2^s and heat flux, 30 W/cm^2^.

To understand the effect of surface properties on bubble dynamics, flow pattern transition and flow regime development in flow boiling microchannels, a series of high-speed visualization studies were employed from the top of both the plainwall and SiNW microchannels. Dielectric fluid HFE-7100 was adopted as the coolant. Frame rate ranges between 4000 and 70,000 fps. Figure 5 represents the time sequence of flow pattern development in flow boiling plainwall microchannels at mass flux, 400 kg/m^2^s and heat flux, 30 W/cm^2^. Plainwall microchannels experiences different flow regimes from nucleate boiling to slug to churn to annular flow throughout its boiling cycle. The cycle starts with rewetting and formation of bubble at the wall. The bubbles do not detach from the wall and form a vapor blanket and later grow and merge with other bubbles to form slugs as time progresses. Afterwards, the slugs merge and grow to form churn flow and evaporation at liquid–vapor interface transforming the churn flow to annular flow. Shortly after the formation of annular flow, rewetting takes place to renew the boiling cycle. The plainwall microchannels take approximately 124 ms to complete the cycle at this condition.

**Figure 6:**
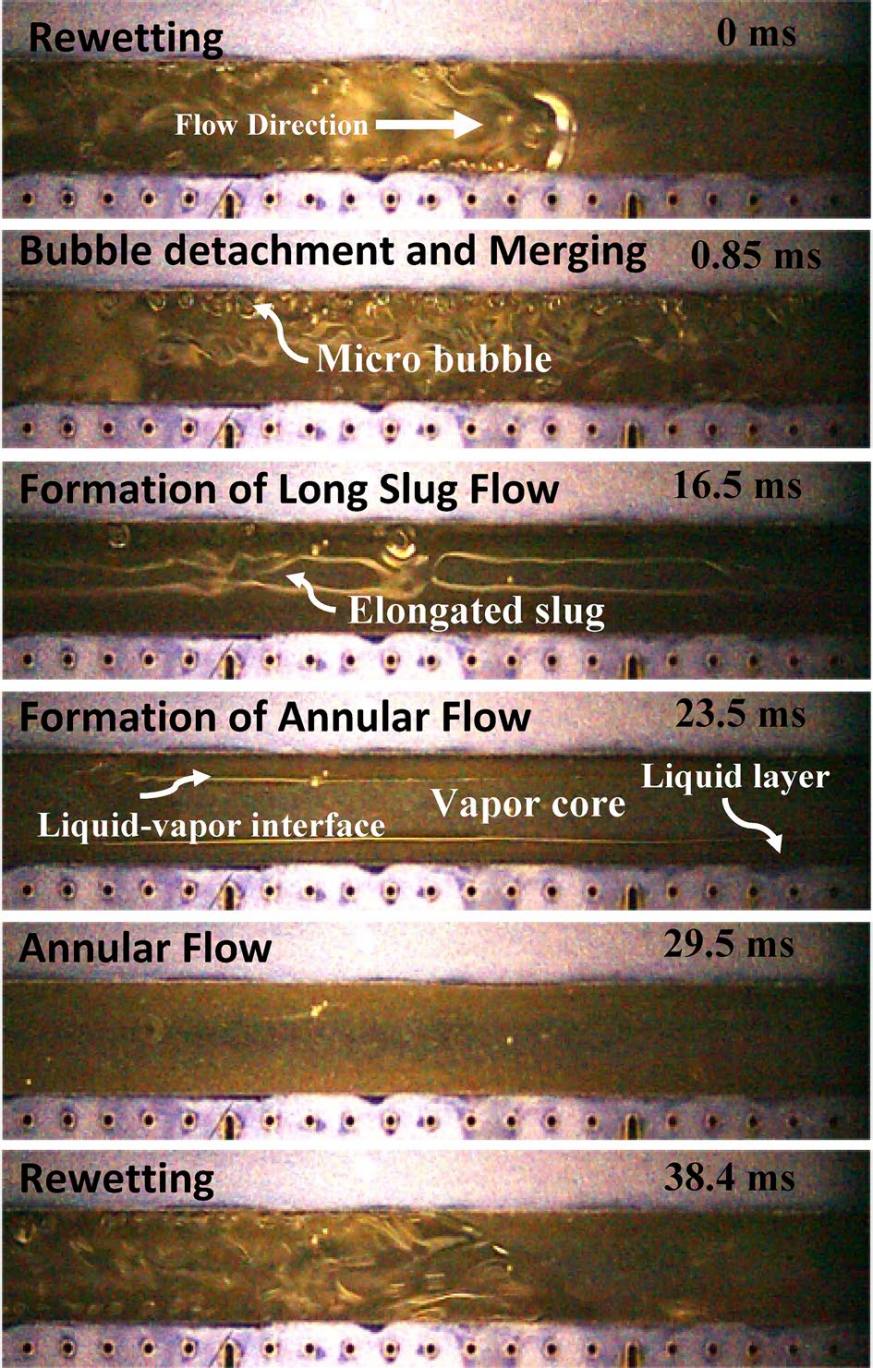
Flow pattern transitions in flow boiling SiNW microchannels at mass flux, 400 kg/m^2^s and heat flux, 30 W/cm^2^.

**Figure 7:**
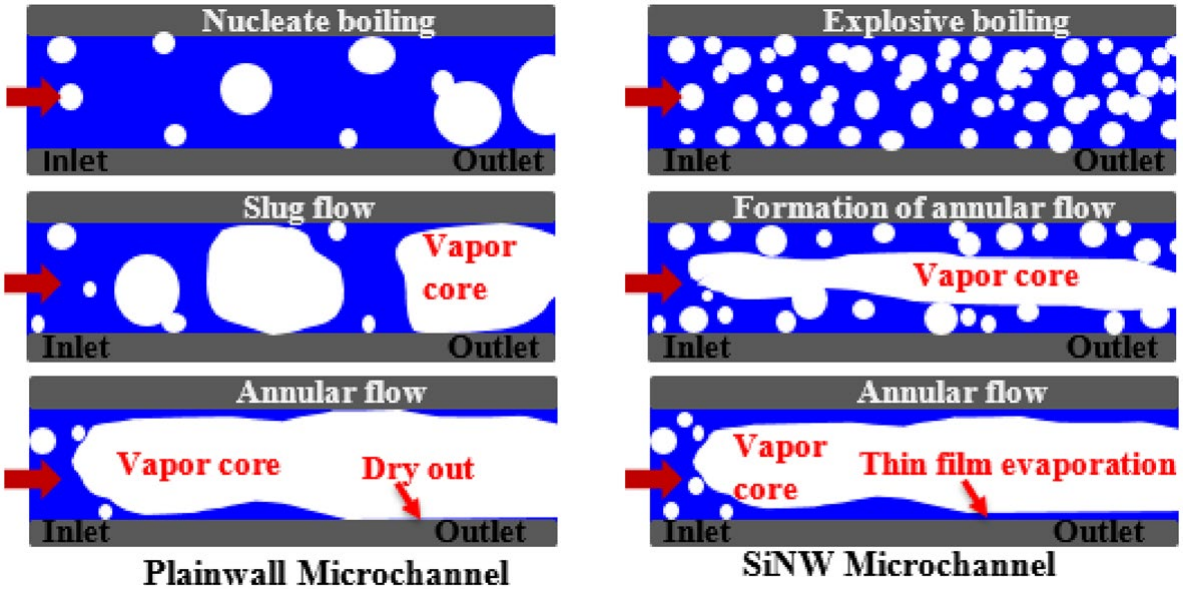
Typical flow pattern transitions of flow boiling in plainwall microchannels and SiNW microchannels.

A different flow pattern transition and flow regime development can be observed in flow boiling SiNW microchannels at the same working conditions and is presented in Fig. 6. Numerous micro bubbles form on the heated wall just after rewetting in SiNW microchannels. The numbers of nucleating bubbles with significantly reduced sizes are much larger in SiNW microchannel compared to plainwall microchannels. Unlike plainwall microchannel, bubbles from SiNW microchannel depart quickly from the heated wall and merge at the middle of the channel to form long vapor slug. Thus, SiNW does not experience vapor blanket on wall and a continuous liquid replenishment on heated wall take places. Evaporation at liquid–vapor interface takes place around the long vapor slug to form complete annular flow and thus no short slug or churn flow are observed in this system. The boiling cycle takes approximately 38 ms, which is three times shorter than plainwall and thus, the formation of annular flow and rewetting are much more frequent (26 Hz in this case) in SiNW microchannels. Therefore, explosive boiling and thin film evaporation dominate in SiNW microchannel. Comparing Figs. 5 and 6, it can be concluded that no vapor blockage is observed due to the absence of intermittent flow patterns (slug/churn flow) and smooth flow pattern transition (from explosive nucleate boiling to annular flow) and flow regulation can be achieved in SiNW microchannel. Thus, SiNW experiences lesser flow resistance and instabilities as reported for plainwall microchannels^40^. A sketch of typical flow pattern transitions in flow boiling plainwall and SiNW microchannels is depicted in Fig. 7 for better understanding.

### Effect of heat and mass flux in SiNW microchannels

**Figure 8:**
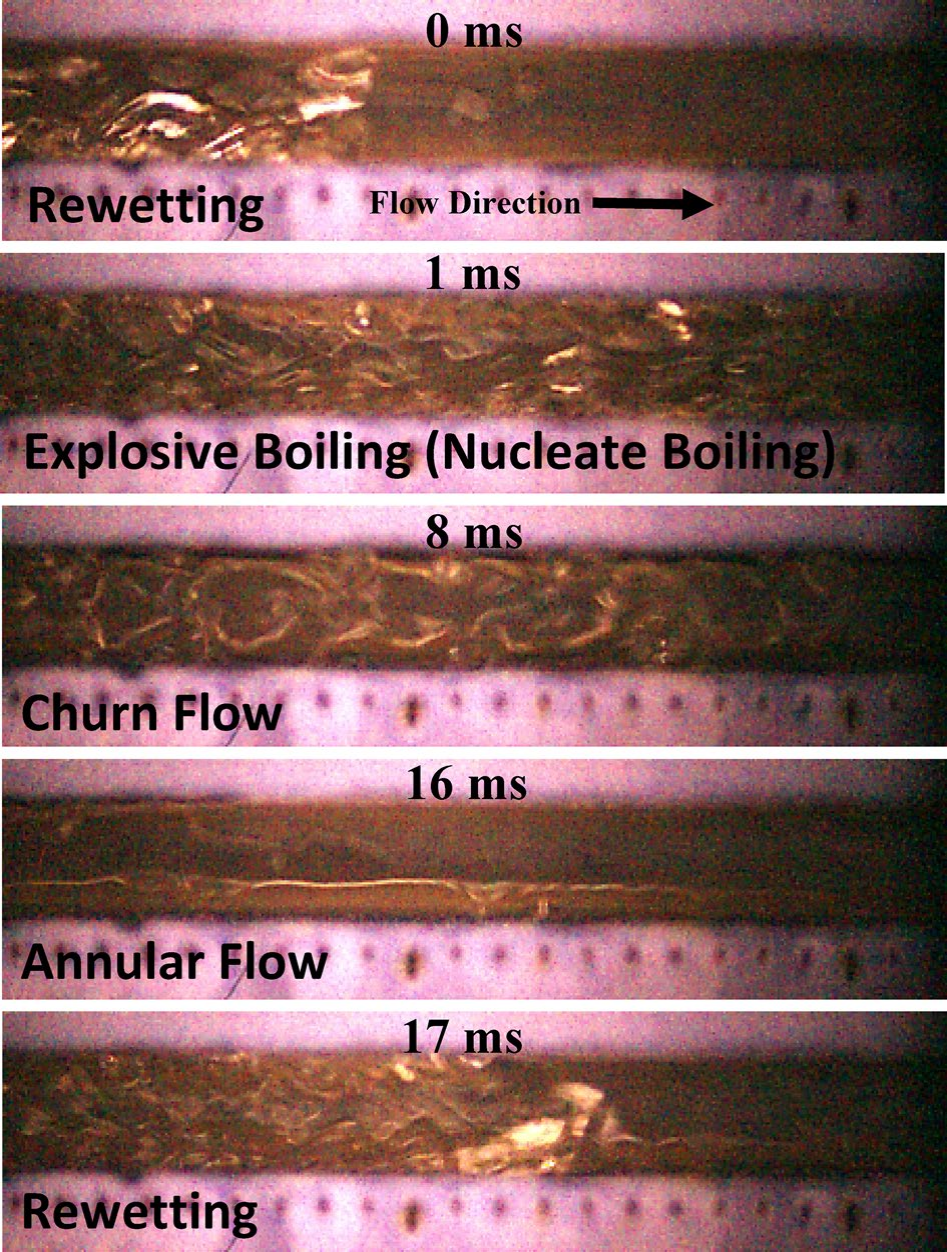
Flow pattern transitions of flow boiling in SiNW microchannels at mass flux, 400 kg/m^2^s and heat flux, 48 W/cm^2^ using dielectric fluid, HFE-7100 at frame rate 50,000 fps.

In the previous section it has been demonstrated that annular flow and thin film evaporation dominates SiNW microchannel (by eliminating intermittent flow regime and having three times shorter flow boiling cycle), which is the most effective regime of flow boiling heat transfer. Therefore, such interesting observation warrants further details analysis to fully understand the effects of heat flux and mass flux on this improved surface. Figure 8 illustrates the high-speed flow boiling visualization pattern sequences in SiNW microchannel at *q*_*eff*_″ = 48 W/cm^2^ and mass fluxes of HFE-7100 400 kg/m^2^s. Comparing Figs. 6 and 8, it can be seen that at higher heat flux condition, more prominent explosive boiling takes places in the SiNW microchannel shortly after the liquid rewetting. Our flow visualization image processing suggests that at mass flux 400 kg/m^2^s and heat flux 30 W/cm^2^, nucleation site density is found to be < 330 bubbles/mm^2^ with bubble departure frequency < 1500 Hz; whereas, at same mass flux but higher heat flux of 48 W/cm^2^, nucleation site density becomes > 400 bubbles/mm^2^ with bubble departure frequency > 4000 Hz. As time progresses, microbubbles from the explosive boiling merge together to form a churn flow pattern. With further advancement of time, churn flow gradually transforms into stable annular flow and then the rewetting appears and the flow boiling cycle repeats. Flow boiling regime like ‘long slug flow’ has not been observed at this high heat flux condition due to numerous microbubble formations termed here as ‘explosive boiling’. Explosive boiling is a process where a superheated liquid undergoes an explosive liquid–vapor phase transition due to massive nucleation of vapor bubbles. Explosive boiling reduces the transitional flow boiling regime and annular flow development time, and hence helps maintaining stable annular flow regime. It also enhances heat transfer performances by enhancing bubble nucleation and thin film evaporation in SiNW microchannel. In addition, the flow boiling cycle duration reduces with the increasing heat flux, i.e., from 38 (for 30 W/cm^2^) to 17 ms (for 48 W/cm^2^).

**Figure 9:**
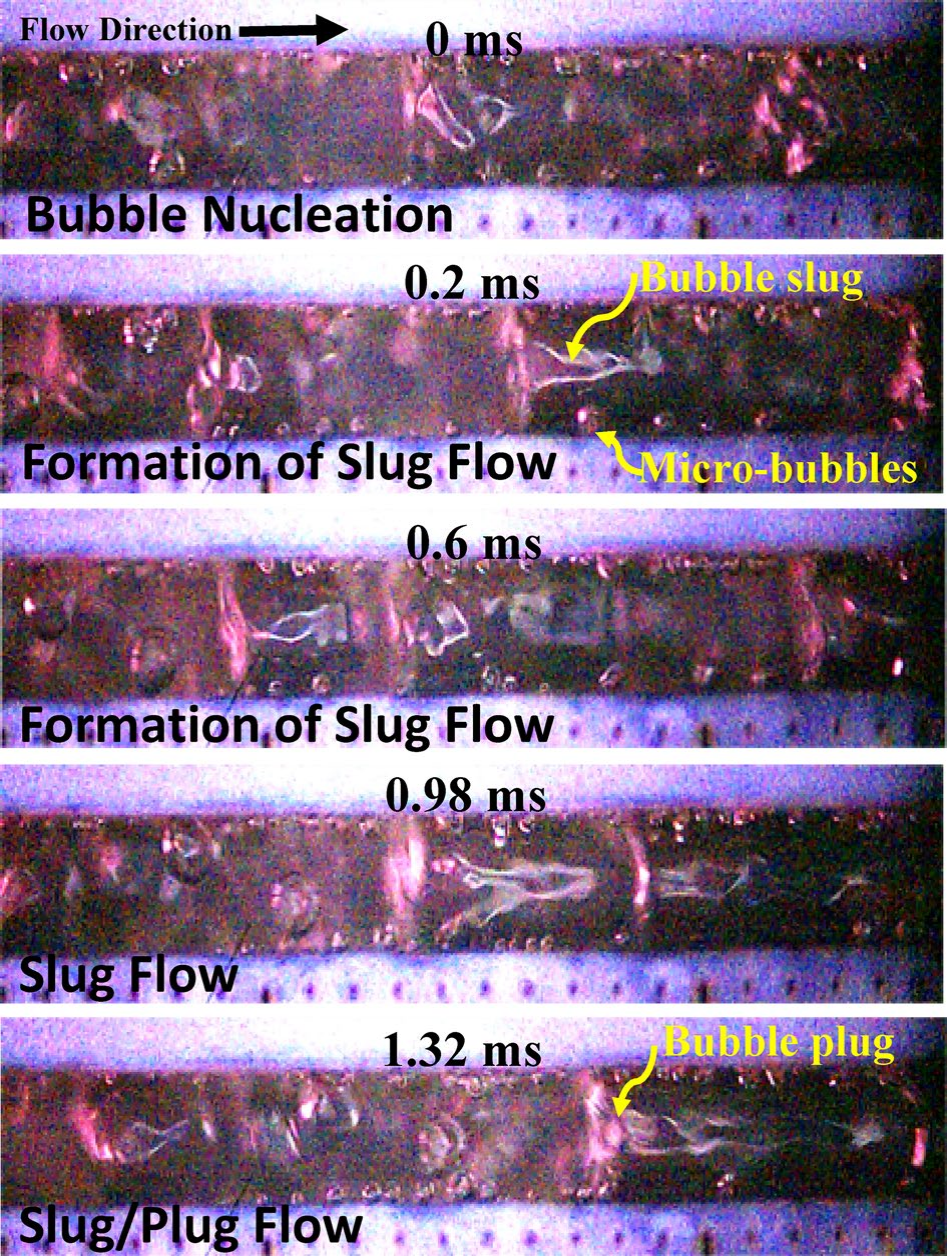
Flow pattern transitions of flow boiling in SiNW microchannels at mass flux, 1000 kg/m^2^s and heat flux, 48 W/cm^2^ using dielectric fluid, HFE-7100 at frame rate 70,000 fps.

**Figure 10:**
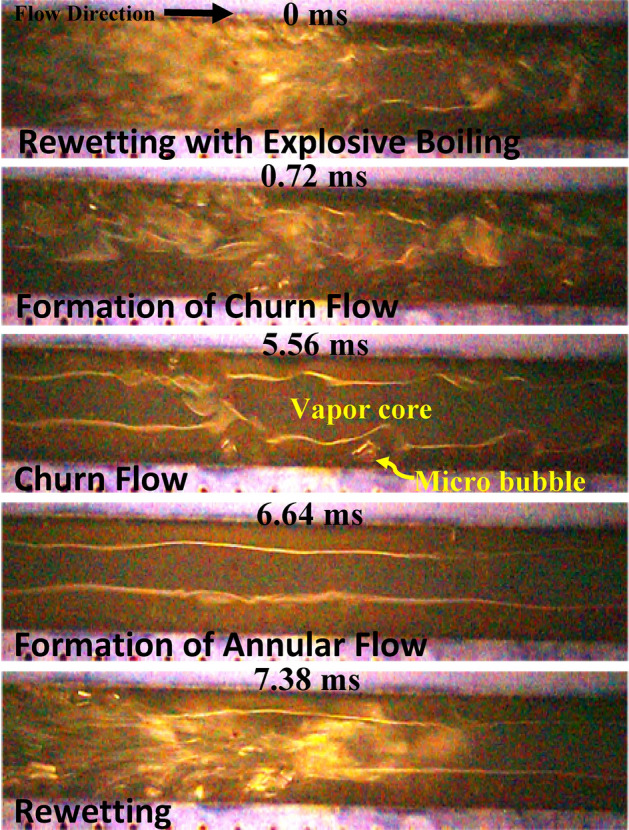
Flow pattern transitions of flow boiling in SiNW microchannels at mass flux, 1000 kg/m^2^s and heat flux, 68 W/cm^2^ using dielectric fluid, HFE-7100 at frame rate 70,000 fps.

Significant differences in flow regime development have been observed from the flow visualization patterns at different mass fluxes, 400 and 1000 kg/m^2^s in SiNW microchannels as shown in Fig. 9. Heat flux was held constant at 48 W/cm^2^. Comparing Figs. 8 and 9, it can be observed from the figures that at same heat flux condition, dominant flow regime shifts from annular flow to slug-plug flow with the increase of mass fluxes from 400 to 1000 kg/m^2^s. This is due to the smaller bubble nucleation density and quick bubble removal process (lesser time to heat transfer and vapor augmentation) at higher mass fluxes. Much shorter flow boiling cycle duration (17 ms for 400 kg/m^2^s versus 1.32 ms for 1000 kg/m^2^s at heat flus 48 W/cm^2^) is observed at higher mass fluxes. However, SiNWs still generate numerous micrometre nucleate bubbles and slug-plug flow is formed at the middle of the channel by merging departing bubbles without blocking the heated wall/ flow channel at this high mass flux condition. With a further increase of heat flux from 48 to 68 W/cm^2^ at higher mass flux condition, 1000 kg/m^2^s; flow boiling regime again shifts from slug-plug flow to churn-annular flow followed by explosive boiling as presented in Fig. 10. At this latter higher mass flux condition, a complete formation of annular flow could not be achieved due to much shorter flow boiling cycle duration. Thus, flow boiling conditions have a significant impact on flow regime development and flow visualization study alone cannot provide a complete picture of the complex two-phase flow pattern transition and flow regime development in this SiNW microchannel.

### Flow boiling characteristics of HFE-7100 in SiNW microchannels

**Figure 11:**
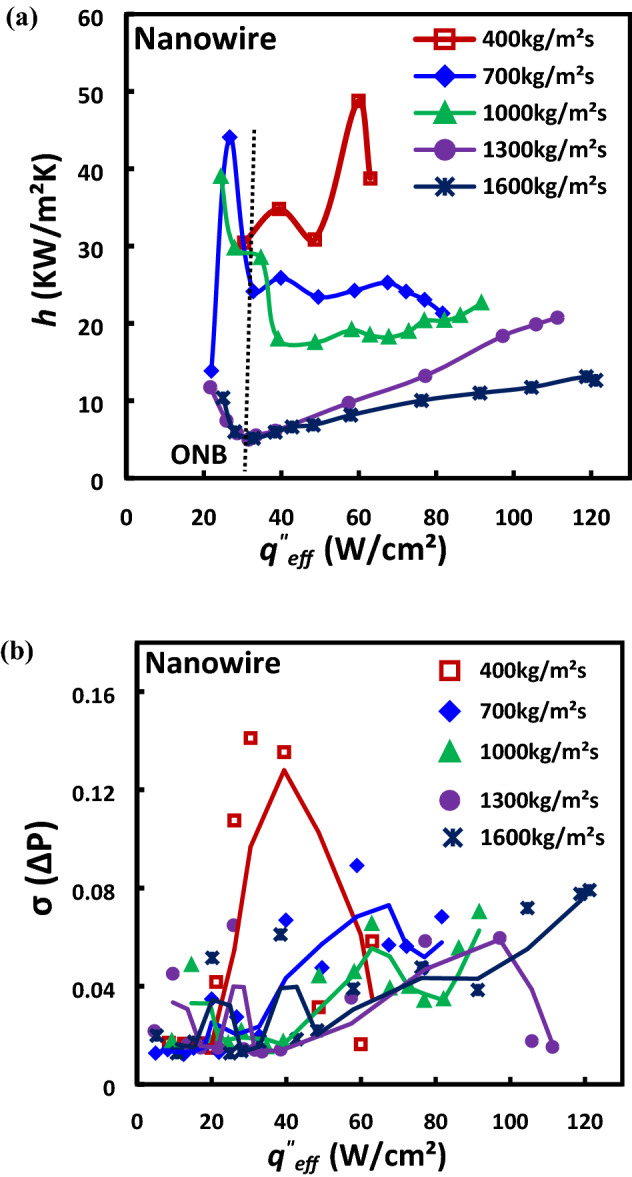
Effects of mass flux in flow boiling SiNW microchannels on (**a**) heat transfer coefficient curve; (**b**) flow boiling instabilities (standard deviation of pressure drop oscillation).

It has been established in our flow visualization studies reported in the previous section that flow regime development and heat transfer mechanism in flow boiling SiNWs microchannels are significantly influenced by operating conditions. Hence, detailed investigation on the effects of heat and mass flux in SiNW are conducted in this section using dielectric fluid, HFE-7100, to understand the boiling phenomena and evaluate the flow boiling SiNW microchannel for practical applications. Figure 11a illustrates the effect of mass flux on the heat transfer coefficient as a function of the effective heat flux for a wide range of mass fluxes from 400 to 1600 kg/m^2^s and heat fluxes up to CHF. The figure suggests that HTC gradually increases after the onset of boiling (ONB) with heat flux at different mass fluxes before reaching to critical heat flux (CHF). This increment in HTC with the increase of heat flux is due to the enhanced nucleate boiling (explosive boiling), followed by a quick transition to annular flow and thin film evaporation throughout liquid–vapor interface. However, after reaching the CHF, HTC gradually decreases due to the local partial dryout and increased interfacial stress at the liquid vapor interface^40^. Higher heat transfer performance is achieved at lower mass flux as can be seen from the figure. At the same heat flux condition, an early transition to annular flow followed by thin film evaporation is achieved at smaller mass flux conditions as observed in the visualization studies which resulted in higher heat transfer coefficient. The heat transfer coefficient gradually decreases with the increase of heat flux shortly after ONB for smaller mass flux conditions up to 700 kg/m^2^s due to the longer flow boiling cycle duration (17 ms for 400 kg/m^2^s compared to 1.32 ms for 1000 kg/m^2^s at same heat flux, 48 W/cm^2^) and stagnant annular flow vapor core with partial dryout zone as can be seen in Fig. 8. Vapor has much lower thermal conductivity than thin liquid film; therefore, heat transfer performances decrease in the system. However, compared to the lower mass fluxes, the heat transfer coefficient gradually increases with the increase of heat flux before reaching CHF at higher mass fluxes above 1000 kg/m^2^s due to the reduced instability resulted from lower vapor quality (reduced channel blockage and reversed flow) and flow regime shift from churn/annular flow to slug flow regime as can be seen by comparing Figs. 8 and 9. Effects of mass flux on flow boiling instabilities (standard deviation of pressure drop oscillation) in flow boiling SiNW microchannel are presented in Fig. 11b. Higher flow boiling instabilities are observed at smaller mass fluxes and flow boiling instabilities reduce with the increase of mass flux as shown in the figure. A drastic shift in boiling curve can be seen in Fig. 11a as mass flux changes from 1000 to 1300 kg/m^2^ s may be due to the boiling regime shift and will discuss further in the following section.

### Flow regime map

**Figure 12:**
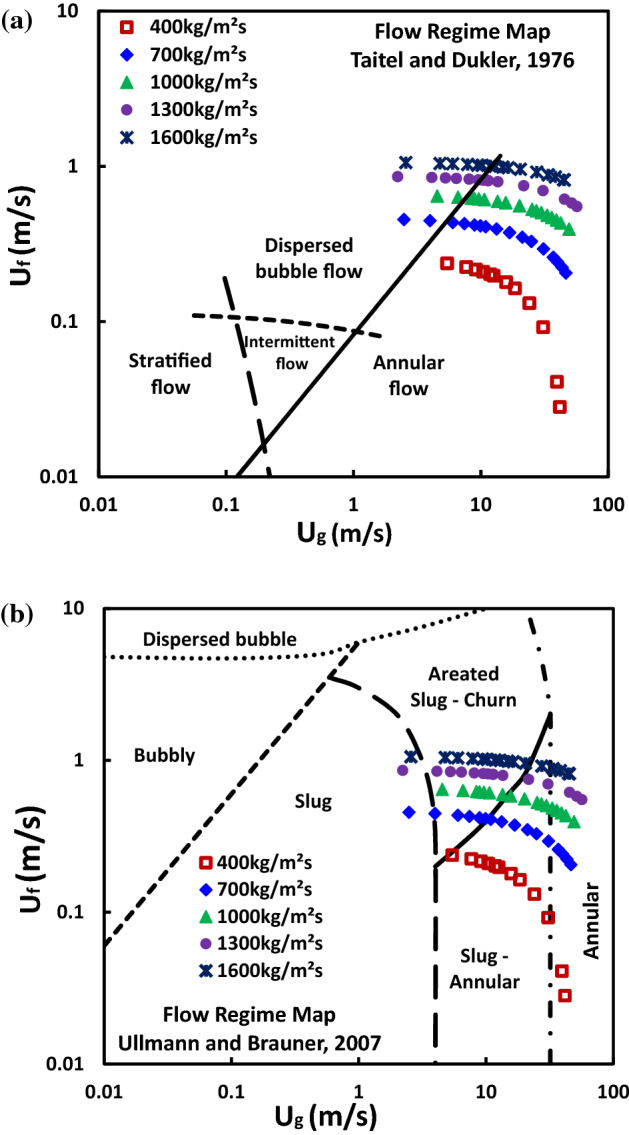
(**a**) Taitel–Dukler^32^ flow regime map for SiNW data (at different mass fluxes of HFE-7100); (**b**) Ullmann–Brauner^33^ flow regime map for SiNW data (at different mass fluxes of HFE-7100).

Flow visualization study alone cannot fully explain and cover the boiling behaviors of SiNWs microchannels at a wide range of flow boiling conditions. In this section, we have studied different flow regime map for SiNW microchannels to better understand the unique feature of flow boiling HFE-7100 in SiNW. The experimental data for SiNW microchannels is plotted on the Taitel–Dukler flow regime map^32^ as shown in Fig. 12a. The Taitel–Dukler flow regime map was developed for adiabatic two-phase flow (no thermal interactions between phases, the pipe, and the environment). The model has proven to be useful in numerous studies and Frankum et al.^41^ have shown experimentally that the methods developed based on adiabatic approach could satisfactorily predict the flow regimes in an evaporating flow. The use of Taitel–Dukler regime map specifically for microchannel flow boiling has also been established by Bar–Cohen and Rahim^42^. The present experimental system is non-adiabatic where all these thermal interactions present. The Taitel–Dukler regime map can provide a reasonable prediction of flow regimes as shown in Fig. 12a. The flow boiling SiNW microchannels experimental data points in Fig. 12a show that most of them are in the annular flow boiling regime and flow regime shifts from dispersed bubble flow to annular flow regime as vapor superficial velocity increases.

Our high speed visualization studies also showed annular flow regime as a dominant flow regime in SiNW microchannels system. However, this flow regime map could not predict the intermittent flow regimes as observed at high mass flux (high superficial velocity of liquid, U_f_) and low heat flux (low superficial velocity of vapor, U_g_) conditions. Though a good agreement of flow regime map with flow visualization studies has been observed using Taitel–Dukler map, a more detail flow regime map proposed by Ullmann–Brauner ^33^ with intermittent flow regimes has been plotted along with our flow boiling experimental SiNW microchannel data in Fig. 12b. Ullmann–Brauner^33^ proposed a flow regime map based on mechanistic model and prediction method for two-phase flow in minichannels, compared with experimental data from literatures and reported a good agreement. Rahim et al.^43^ has shown that this method could predict slug and annular flow regimes satisfactorily. In present study, flow boiling SiNW experimental data has been plotted in Ullmann–Brauner regime map and it can be seen from the Fig. 12b that annular flow regime is still dominated for smaller mass flux. However, flow regime shifts from Slug-Annular to Aerated Slug—Churn as mass flux increases and from annular to slug as heat flux decreases. Our current flow visualization studies show excellent agreement with this flow regime map as can be seen from Figs. 6, 7, 8, 9 and 10 and thus, this map can be used to predict the flow boiling HFE-7100 in SiNW microchannels characteristics for a wide range of operating conditions.

A satisfactory prediction of flow regime and flow regime transition of Flow boiling HFE-7100 in SiNW microchannel can be achieved from these two flow regime maps. However, flow boiling behavior in microchannel differs from mini/macrochannel and to correctly predict the unique flow regime development and transition in these microscale geometries, a more comprehensive flow regime map presented by Harirchian and Garimella^34, 35^ is adopted in this study. This flow regime map can accurately explain the behavior (a drastic shift in boiling curve as mass flux changes from 1000 to 1300 kg/m^2^s) as observed in Fig. 11a.

**Figure 13:**
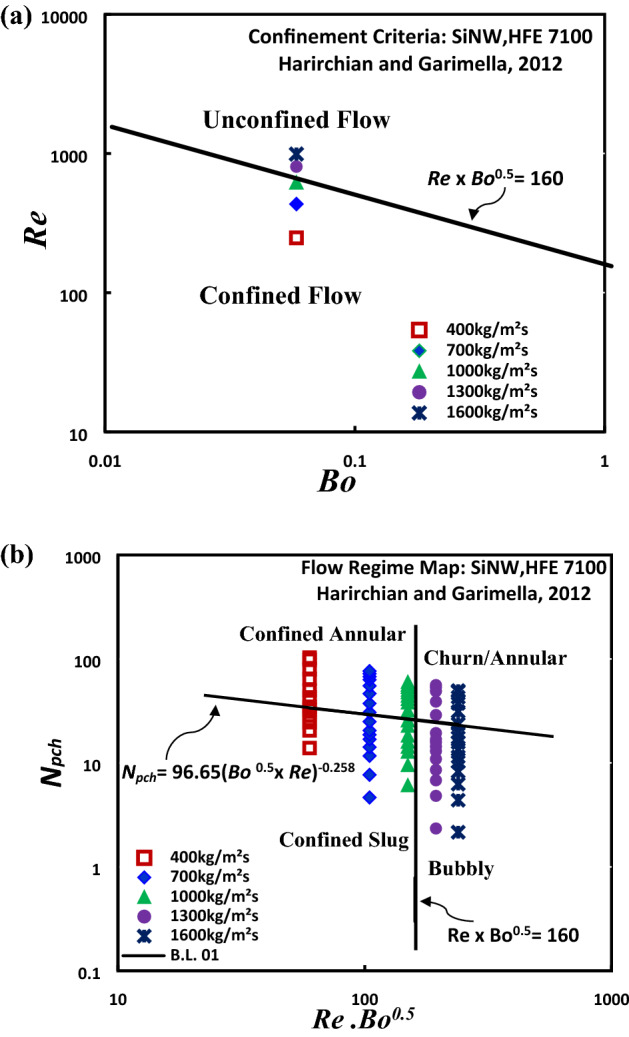
(**a**) Vapor confinement criteria at different mass fluxes of HFE 7100 and (**b**) Harirchian and Garimella^34, 35^ flow regime map for SiNW at different mass fluxes of HFE-7100.

Vapor confinement criteria in SiNW microchannels at different mass fluxes are illustrated in Fig. 13a. The confinement criterion based on dimension, fluid properties and mass flux as described by Harirchian and Garimella^34, 35^ is used in this work.

Bond number is defined as the ratio of buoyancy force to surface tension force.

1 $$Bo = \left[ {\frac{{g(\rho_{f} - \rho_{g} )}}{\sigma }} \right]{\text{D}}_{{\text{h}}}^{2}$$

where σ is the surface tension, g is the gravitational acceleration, $\rho_{f}$ and $\rho_{g}$ are liquid and vapour densities of fluid respectively. $D_{h}$ is the channel hydraulic diameter. Some other non-dimensional parameter like Reynolds number, *Re* are defined as follows:

2 $$Re = \frac{{GD_{h} { }}}{{\mu_{f} }}$$

This flow boiling transition criterion recommends that for $Bo^{0.5} \cdot\,Re < 160$, vapour bubbles are confined in microchannels. It is clearly noted from the Fig. 13a that SiNW microchannels show transformation from confined to unconfined flow as mass flux increases from 1000 kg/m^2^s to higher. Confined flow promotes higher heat transfer performance due to thin film evaporation and heat transfer performance deteriorates in unconfined flow. As flow boiling behavior shift from confined to unconfined flow above 1000 kg/m^2^s, a shift in heat transfer performance is observed as shown in Fig. 11a.

Harirchian and Garimella^34, 35^ have shown that flow confinement depends not only on the channel dimensions and fluid properties but also on the mass and heat flux since the bubble diameter varies with flow rate and heat flux. Harirchian and Garimella^34, 35^ developed a flow regime map by correlating the convective confinement number, $(Bo^{0.5} \cdot\,Re$) and phase change number, *N*_*pch*_· The phase change number represents the rate of phase change due to heat addition and is defined as,

3 $$N_{pch} =\Omega \tau$$

where $$\Omega = \frac{{q^{\prime\prime}_{w} P_{H} \left( {\rho_{f} - \rho_{g} } \right)}}{{A_{c} h_{fg} \rho_{f} \rho_{g} }}$$ is the frequency of vapor generation and $${\uptau } = \frac{{L_{H} }}{{{\raise0.7ex\hbox{$G$} \!\mathord{\left/ {\vphantom {G {\rho_{f} }}}\right.\kern-\nulldelimiterspace} \!\lower0.7ex\hbox{${\rho_{f} }$}}}}$$ is the fluid particle residence time. $$P_{H}$$ is the heated channel perimeter, $$L_{H}$$ is the heated length and $$A_{c}$$ is the channel cross-sectional area. Based on this, a new transition line has been proposed,

4 $$N_{pch} = 96.65\left( {{\text{Bo}}^{0.5} \times {\text{ Re}}} \right)^{ - 0.258}$$

By adopting this method, Fig. 13b presents the flow regime map. It can be seen from the figure that vapor confinement is observed for low range of mass flux and as heat flux increase, transition occur from confined slug to confined annular flow.

### Enhanced flow boiling in SiNW microchannels

**Figure 14:**
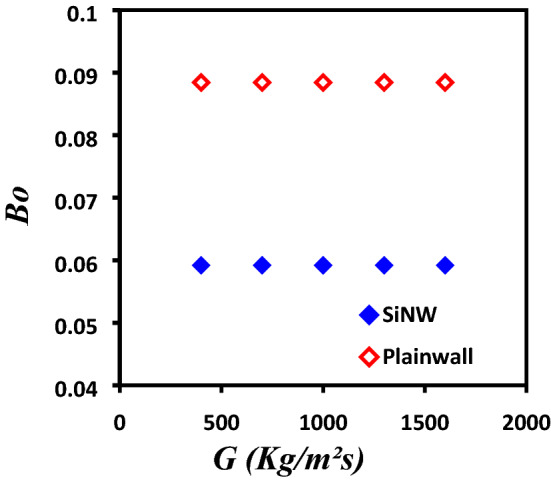
Comparison of Bond number, Bo between silicon nanowire and plainwall microchannels.

It has been established in our earlier studies^27, 31^ that SiNWs enhances flow boiling microchannel system performances significantly compare to plainwall microchannels using deionized water as working fluid. However, low latent heat of evaporation and poor surface tension of dielectric fluid, e.g. HFE-7100, could significantly alter the SiNWs heat transfer performance. Hence, further studies were necessary to compare the flow boiling characteristics between Plainwall and SiNW microchannels. Bond number, *Bo* (the ratio of body force to surface tension force) is plotted as a function of mass flux, *G* as shown in Fig. 14. It can be seen that *Bo* is higher for plainwall microchannel compared to SiNW; hence body force (gravitational effect) has more impact on plainwall microchannel. Therefore, SiNW is more gravity insensitive and suitable for practical microgravity applications.

**Figure 15:**
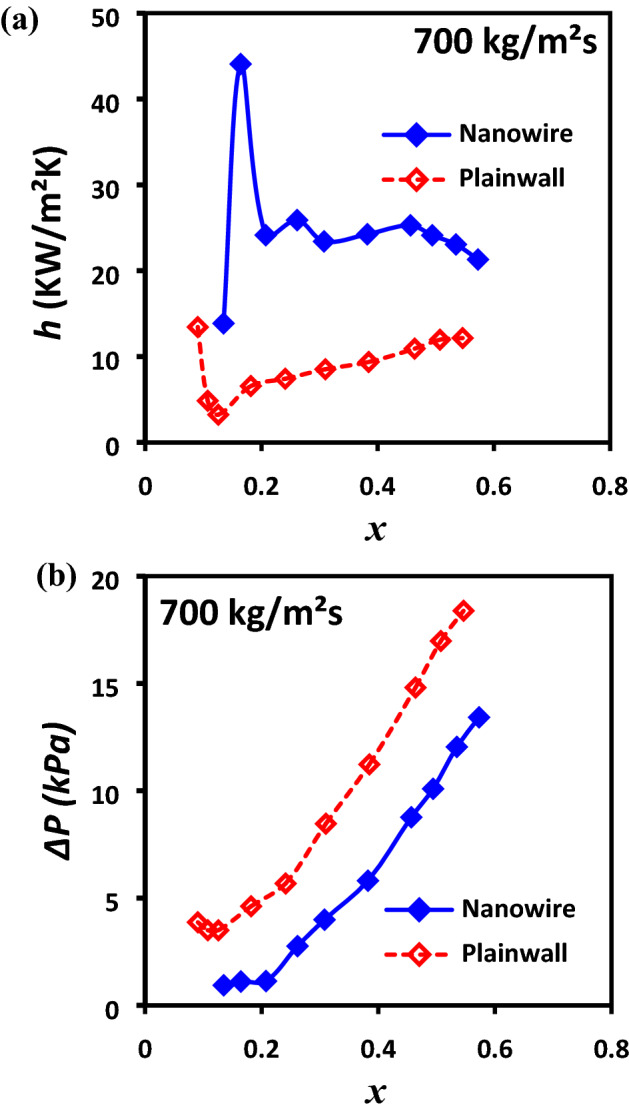
Comparison of (**a**) heat transfer coefficient, h; (**b**) pressure drop, ∆P as a function of vapor quality between silicon nanowire and plainwall microchannels.

Heat transfer coefficients, h, for Silicon Nanowire (SiNW) and plainwall microchannels have been plotted as a function of vapor quality at mass flux, 700 kg/m^2^s and reported in Fig. 15a. Heat transfer coefficients (HTC) gradually increases with the vapor quality in SiNW microchannel before reaching to CHF. These enhancements in SiNW at lower vapor quality are due to the dominant stable annular flow regime and thin film evaporation at vapor–liquid boundary layers throughout the entire channel. However, the HTC gradually decreases for SiNW at higher vapor quality due to the local partial dryout and increased interfacial stress at liquid vapor interface^40^. An improved enhancement in HTC has been found for SiNW compare to plainwall microchannels. For example, HTC was enhanced up to 176% from 8.5 kW/m^2^K in plainwall to 23.5 kW/m^2^K in SiNW at same vapor quality and mass flux condition, 0.3 and 700 kg/m^2^s respectively. This enhanced heat transfer performance in SiNW microchannels is due to the increased bubble nucleation (explosive boiling), reduction of transitional flow regime, quick formation of stable annular flow regime and extended thin film evaporation as discussed early in the flow visualization studies by comparing Figs. 5 and 6. To the contrary, bubble in plainwall microchannel cannot maintain thin film, leading to dry patch and eventually heated wall comes in contact with the vapor film. Since vapor has much lower thermal conductivity than thin liquid film, heat transfer performances deteriorate in the plainwall system. However, unlike plainwall microchannel, SiNWs microchannel does not create vapor blanket on the heated wall due to the quick bubble removal process and frequent liquid renewal resulting in enhanced heat transfer performances.

Comparison of pressure drop, *∆P* as a function of vapor quality between SiNW and plainwall microchannels at mass flux, 700 kg/m^2^s is presented in Fig. 15b. The figure illustrates that pressure drop increases with the vapor quality due to the increased acceleration effect of vapor content for both the plainwall and SiNW microchannel. In addition, SiNW maintains much smaller pressure drop compare to plainwall due to the stable annular flow dominance and excellent two-phase separation as shown and compared in Figs. 5 and 6. In plainwall microchannel, unstable and irregular vapor–liquid interface creates higher frictional losses which resulted in greater pressure drop. On the other hand, smooth flow transition and undisturbed liquid–vapor interface, as presented in the visualization results analysis section, reduces the frictional loss and thus the overall lower pressure drop in the SiNW microchannels.

## Conclusions

In summary, the unique flow regime formation, transitional phenomena and bubble dynamics of flow boiling HFE-7100 in SiNW microchannels are reported and compared with the conventional plainwall microchannel. High-speed flow visualization up to 70,000 fps has been utilized along with the extensive experimental investigations to understand the boiling mechanisms (i.e., bubble dynamics, flow regulation, flow patterns and flow regime developments) and system performances (in terms of heat transfer rate, pressure drop and instability characteristics) in SiNW microchannels. Furthermore, SiNW experimental data have been plotted in different established flow regime maps (Taitel–Dukler map^32^, Ullmann–Brauner^33^, Harirchian and Garimella^34, 35^) to better understand the observed flow patterns during visualization studies. Excellent agreements have been observed between experimental results and flow regime map. Major differences in flow boiling performances, bubble dynamics and flow pattern developments have been observed at different mass flux conditions in SiNW microchannels and explosive boiling and annular flow dominated in these systems. In addition, favourable flow regulation was achieved in flow boiling HFE-7100 SiNW microchannel by enhancing liquid renewal and nucleate boiling (explosive boiling), reducing bubble departure diameter, smoothing flow transition, increasing thin film evaporation by flow separation and by minimizing intermittent flow regimes which resulted excellent improved system performances.

The physical insight this study provided on the flow boiling in SiNW microchannels on flow regulations and performance enhancements using dielectric fluids can pave the way for development of next generation high performance gravity insensitive two-phase heat sinks for space applications.

## Supplementary Information

Supplementary Information.
