# Supplementary material for: Favourably regulating two-phase flow regime of flow boiling HFE-7100 in microchannels using silicon nanowires
Source: Sci Rep. 2021 May 27;11:11131. doi: 10.1038/s41598-021-89466-z (PMC8160198; doi:10.1038/s41598-021-89466-z)
Supplement: Supplementary file 1 — Supplementary Information. [file 41598_2021_89466_MOESM1_ESM.docx]

**Favourably Regulating Two-phase Flow Regime of Flow Boiling HFE-7100 in Microchannels using Silicon Nanowires**

Tamanna Alam^1^, Wenming Li^1^, Wei Chang^1^, Fanghao Yang^2^, Jamil Khan^1^, Chen Li^1,*^

*^1^Department of Mechanical Engineering, University of South Carolina, Columbia, SC, 29210, United States*

^2^Princeton Plasma Physics Laboratory, Princeton, NJ, 08540, United States

^*^Corresponding author. Tel: +1 803 777 7155; Email address: [li01@cec.sc.edu](mailto:li01@cec.sc.edu)

**Supplementary Materials**

**Data Reduction.** The effective heat transfer rate, $q_{eff}$ to the fluid in SiNW microchannel is obtained by:

$q_{eff}=q-q_{loss}$……………………………..…… (1)

where$q$ is input power ($q=V\times I$) and $q_{loss}$ is heat loss during flow boiling experiment.

The effective heat flux$q_{eff}^{"}$ that the heat sink can dissipate is calculated from:

$q_{eff}^{"}=\frac{q_{eff}}{A}$ ……………………………………..... (2)

where *A* is the heating area of the test chip, *A=W × L* and *W* and *L* are the width and length of the chip.

The average wall temperature, $T_{w}$ is corrected assuming one dimensional heat conduction through the substrate.

$T_{w}=T_{heater}-\frac{q_{eff}^{"}t_{w}}{K_{s}}$ .................................................... (3)

where $t_{w}$ and $K_{s}$ are the substrate wall thickness and thermal conductivity respectively.$T_{heater}$ is the average temperature of the thin film heater (i.e. the thermistor) and is calculated as:

$T_{heater}=K\left( R-R_{a} \right)+T_{a}$ .............................................. (4)

where *R* is the resistance of the micro heater, $R_{a}$ is the resistance of the micro heater at ambient temperature $T_{a}$ and *K* is the slope of the heater electrical resistance-temperature calibration curve.

The heat transfer coefficient in SiNW microchannel is calculated from,

$h =\frac{q_{eff}}{N(wL+2\eta HL)(T_{w-}T_{f})}$………………………………………………........… (5)

where *N* is total number of channels; $w$, $H$ and L are the width, depth and length of the channel respectively and $\eta$is the efficiency of a fin with adiabatic tip which is correlated by:

$\eta= \frac{\tanh(mH)}{mH}$ .................................................................................... (6)

and

$m=\sqrt{\frac{2h}{K_{s}w_{w}}}$........................................................................................ (7)

where $K_{s}$ is the thermal conductivity of the substrate and $w_{w}$ is the width of the channel wall.

$T_{f}$is the fluid temperature as defined by

$T_{f}=T_{f,i}+\frac{q_{eff}^{''}Wz}{\dot{m}c_{p}}$(Single-phase region) ………... (8)

where $T_{f,i}$, $z$, $\dot{m}$ and $c_{p}$ are the subcooled inlet fluid temperature, axial distance, mass flow rate and specific heat respectfully.

$T_{f}=T_{sat}$(Saturated region) ………………….….. (9)

$T_{sat}$ is a function of working pressure, *P*, in the middle of a microchannel, which is estimated as:

$P=P_{i}-\frac{\Delta P}{2}$ ………………….….. (10)

where $P_{i}$ is the inlet pressure; and $\Delta P$ is the pressure drop.

The exit vapor quality is calculated with mass flow rate and input power and expressed as:

$x_{e}=\frac{q_{eff}-q_{subcooled}}{\dot{m}h_{fg}}$ ………………….….. (11)

where, $\dot{q_{subcooled}=\dot{m}}c_{p}(T_{sat-}T_{f,i})$ ………………….….. (12)

Further details of data reduction can be found in Yang et al. [^27^](#_ENREF_27)^,^[^31^](#_ENREF_31).

The measurement accuracies and experimental uncertainties associated with sensors and parameters are listed in Table 2. Uncertainties of measured values are adopted from the manufacturers’ specification sheets, and the uncertainties of derived variables are calculated according to the propagation of uncertainty analysis [^44^](#_ENREF_44). Repeatability of the experimental data has been carried out for different mass fluxes, heat fluxes, orientations and test samples extensively for our current test setup and found within ±2% [^28^](#_ENREF_28).

| **Sensors and Parameters** | **Accuracies and Uncertainties** |
| --- | --- |
| Thermocouple | ±0.5 °C |
| Mass flux, *G* | 2% |
| Pressure drop, $\Delta P$ | 0.4% |
| Voltage on the heater, *V* | 0.10% |
| Current on the heater, *I* | 0.10% |
| Temperature, *T* | 0.8 °C |
| Electrical resistance, *R* | 0.20% |
| Heat flux | 0.30% |
| Heat transfer coefficient | 4% |

**Table 2.** Accuracies and Uncertainties of key parameters (estimated at the working condition: G = 700 kg/m^2^s and $q_{eff}^{''}=$ 60 W/cm^2^)
